# Development and Validation of a Machine Learning-Based Radiomics Model on Cardiac Computed Tomography of Epicardial Adipose Tissue in Predicting Characteristics and Recurrence of Atrial Fibrillation

**DOI:** 10.3389/fcvm.2022.813085

**Published:** 2022-03-03

**Authors:** Min Yang, Qiqi Cao, Zhihan Xu, Yingqian Ge, Shujiao Li, Fuhua Yan, Wenjie Yang

**Affiliations:** ^1^Department of Radiology, Ruijin Hospital, Shanghai Jiao Tong University School of Medicine, Shanghai, China; ^2^Siemens Healthineers Computed Tomography (CT) Collaboration, Shanghai, China

**Keywords:** atrial fibrillation, computed tomography angiography, epicardial adipose tissue, radiomics approach, recurrence

## Abstract

**Purpose:**

This study aimed to evaluate the feasibility of differentiating the atrial fibrillation (AF) subtype and preliminary explore the prognostic value of AF recurrence after ablation using radiomics models based on epicardial adipose tissue around the left atrium (LA-EAT) of cardiac CT images.

**Method:**

The cardiac CT images of 314 patients were collected wherein 251 and 63 cases were randomly enrolled in the training and validation cohorts, respectively. Mutual information and the random forest algorithm were used to screen for the radiomic features and construct the radiomics signature. Radiomics models reflecting the features of LA-EAT were built to differentiate the AF subtype, and the multivariable logistic regression model was adopted to integrate the radiomics signature and volume information. The same methodology and algorithm were applied to the radiomic features to explore the ability for predicting AF recurrence.

**Results:**

The predictive model constructed by integrating the radiomic features and volume information using a radiomics nomogram showed the best ability in differentiating AF subtype in the training [AUC, 0.915; 95% confidence interval (CI), 0.880–0.951] and validation (AUC, 0.853; 95% CI, 0.755–0.951) cohorts. The radiomic features have shown convincible predictive ability of AF recurrence in both training (AUC, 0.808; 95% CI, 0.750–0.866) and validation (AUC, 0.793; 95% CI, 0.654–0.931) cohorts.

**Conclusions:**

The LA-EAT radiomic signatures are a promising tool in the differentiation of AF subtype and prediction of AF recurrence, which may have clinical implications in the early diagnosis of AF subtype and disease management.

## Introduction

Atrial fibrillation is the most common arrhythmia (1). Common complications of AF include: heart failure, coronary heart disease, stroke, etc., resulting in a fatality rate of about 20% and a disability rate of nearly 60% (2–5).

AF can be characterized as paroxysmal and persistent according to the duration of their episodes. The characterization of patients with AF has clinical relevance in the treatment strategy and therapy outcomes. Clinical studies have confirmed the effectiveness and safety of catheter ablation in the treatment of AF. However, persistent AF (PeAF) remains burdened with higher recurrence rates after catheter ablation than paroxysmal AF (PAF) (6, 7). Therefore, accurate AF subtype characterization is essential for patient evaluation and prognosis prediction.

Meanwhile, epicardial adipose tissue (EAT) is a special visceral adipose tissue located between the myocardium around the heart and the visceral pericardium. It reflects visceral obesity and is a crucial source of inflammatory mediators (8–10). EAT surrounding the left atrium (LA-EAT) may cause local inflammation and fibrosis (8, 10, 11). Mazurek et al. found that LA-EAT has higher inflammatory activity compared to subcutaneous or visceral thoracic tissues (12).

EAT can be assessed by using non-invasive imaging techniques especially on cardiac computed tomography (CT) with high spatial resolution. It is reported that the EAT volume around the whole heart was found to be correlated with the AF occurrence, severity, and recurrence (13–15). Furthermore, increased fat thickness directly adjacent to the LA is notably related to the AF burden (16). Ciuffo et al. found that the CT attenuation of the LA-EAT was an independent predictor of AF recurrence after the first ablation (17). The morphological and quantitative analysis of both EAT and LA-EAT showed potential in differentiating AF characteristics and predicting recurrence.

Radiomics has recently received increased attention in medical imaging analysis. Radiomics can quantitatively describe tissue heterogeneity, which was objective but not visually recognizable, by extracting quantitative features from medical images with high throughput (18–20). In the cardiovascular imaging field, radiomics has been proved useful in several topics, such as differentiating the myocardium of hypertensive heart disease from hypertrophic cardiomyopathy (21), identifying coronary plaques with napkin-ring sign, etc. (22). These studies showed that radiomics may have the potential to provide an accurate prediction for the AF subtype characteristics and recurrence, given its broad application prospects.

Therefore, this study aims to analyze radiomic features extracted from the LA-EAT on cardiac CT images and establish a machine learning-based radiomics model to differentiate the characteristics and further predict AF recurrence after ablation, which is expected to provide guidance to identify high-risk individuals (PeAF and AF recurrence after ablation), to actively intervene in treatment to improve patient prognosis.

## Materials and Methods

### Study Population

**Figure 1:**
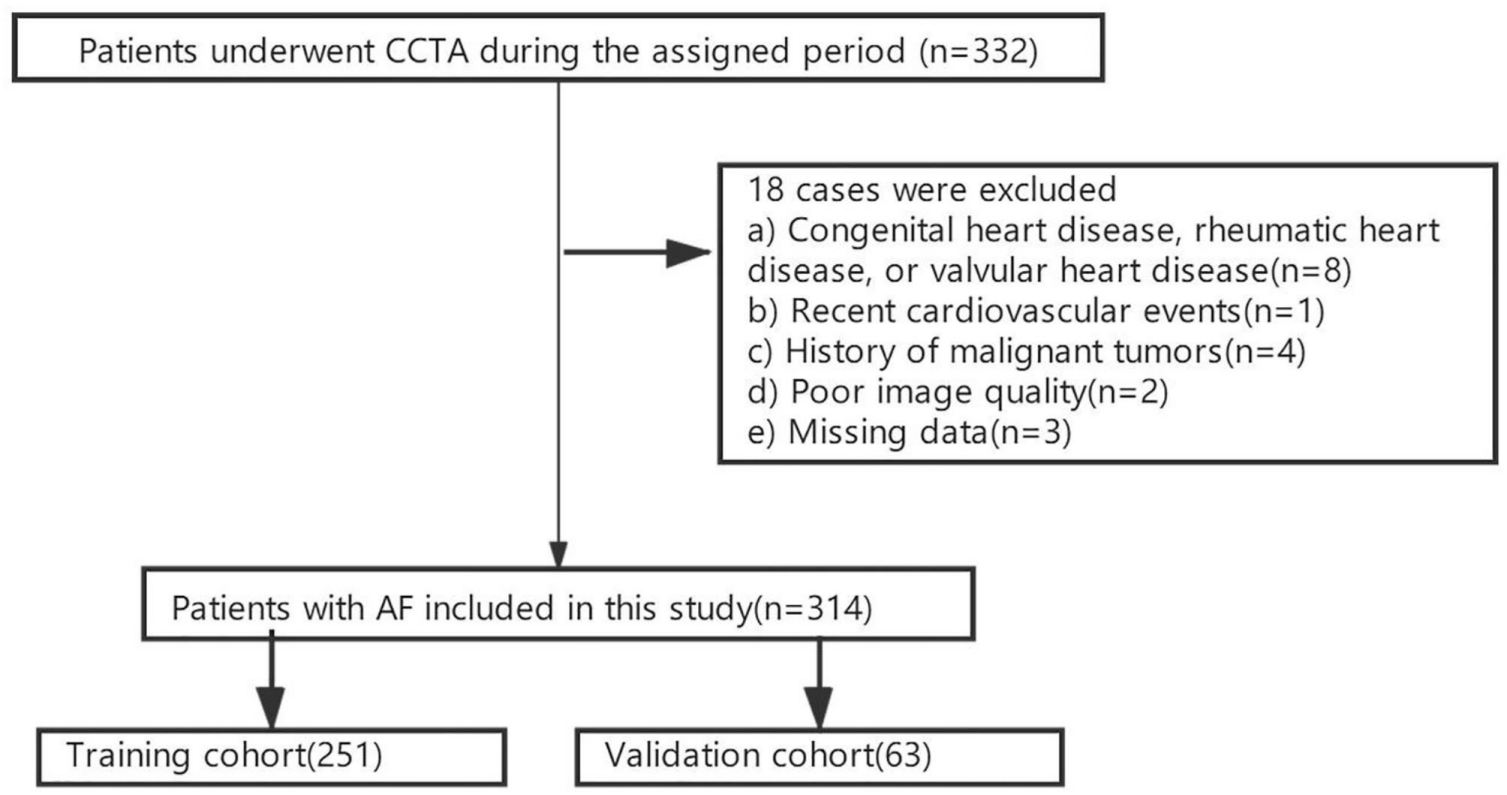
The patient enrollment flow diagram.

**Figure 2:**
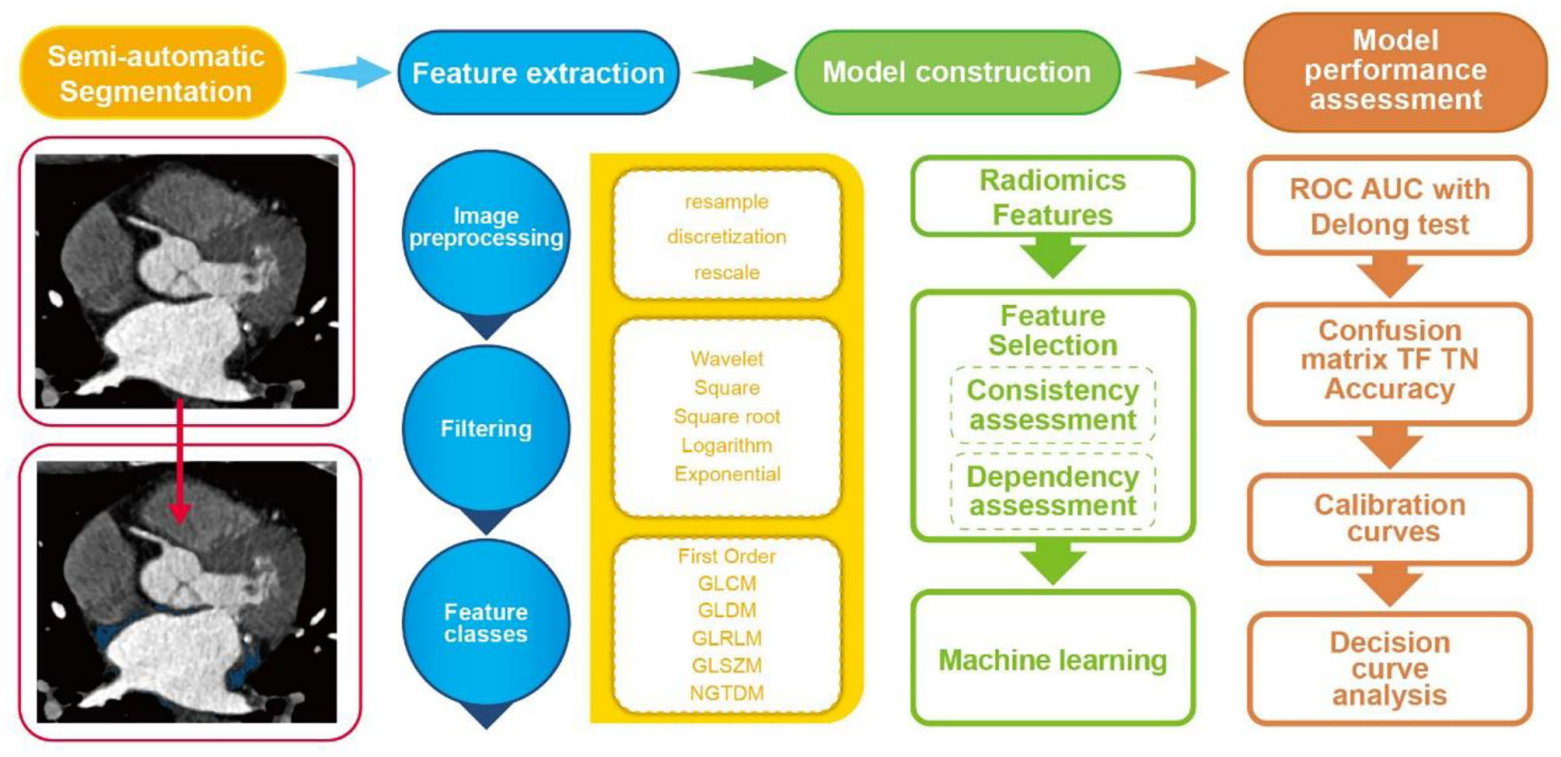
Flowchart of the current study.

This retrospective study was approved by the Institutional Review Board. The written informed consent was waived. The population of this study consisted of 332 hospitalized AF patients who underwent cardiac computed tomography angiography (CCTA) of LA and pulmonary veins (PV) from January 1st 2017 to December 31th 2017 were enrolled. All our patients received ablation as a treatment procedure. According to the exclusion criteria, 314 patients with non-valvular AF (207 and 107 patients with PAF and PeAF, respectively) were finally enrolled in this study (Supplementary Data 1). The flow diagram of this study is illustrated in Figure 1. The cohort was then randomly divided into the training and validation cohorts of 251 and 63 patients, respectively, at an 8:2 ratio (Figure 2).

### Ablation Procedure

The patient was treated with cryo-balloon ablation of AF and local anesthesia with 2% lidocaine. Subsequently, the left and right femoral veins were punctured, and electrodes were placed in the coronary sinus, right ventricular apex, and superior vena cava. Complete atrial septal puncture under the guidance of X-rays to assess the shape, thickness, and presence of branches and co-intervention of PVs. The guide wire is inserted into the LA, and then place the cryo-balloon delivery catheter to deliver the cryo-balloon, and cryo-balloon ablation is performed on the four PVs. The ablation endpoint was defined as the electrical isolation of bilateral PV.

### Follow-Up

All patients received 8 weeks of antiarrhythmic medication after AF catheter ablation. AF recurrence was defined as AF, atrial flutter, or atrial tachycardia that were recorded on the electrocardiograph or 24 h dynamic electrocardiograph for more than 30 s after the blank period of ablation (within the first 3 months after surgery) (23) within 1 year.

### Image Acquisition and EAT Morphological Measurement

All CCTA scanning was accomplished using a third-generation dual-source CT scanner (Somatom Force, Siemens Heathineers, Forchheim, Germany). The detailed scanning protocol and image acquisitions are shown in Supplementary Data 2.

**Figure 3:**
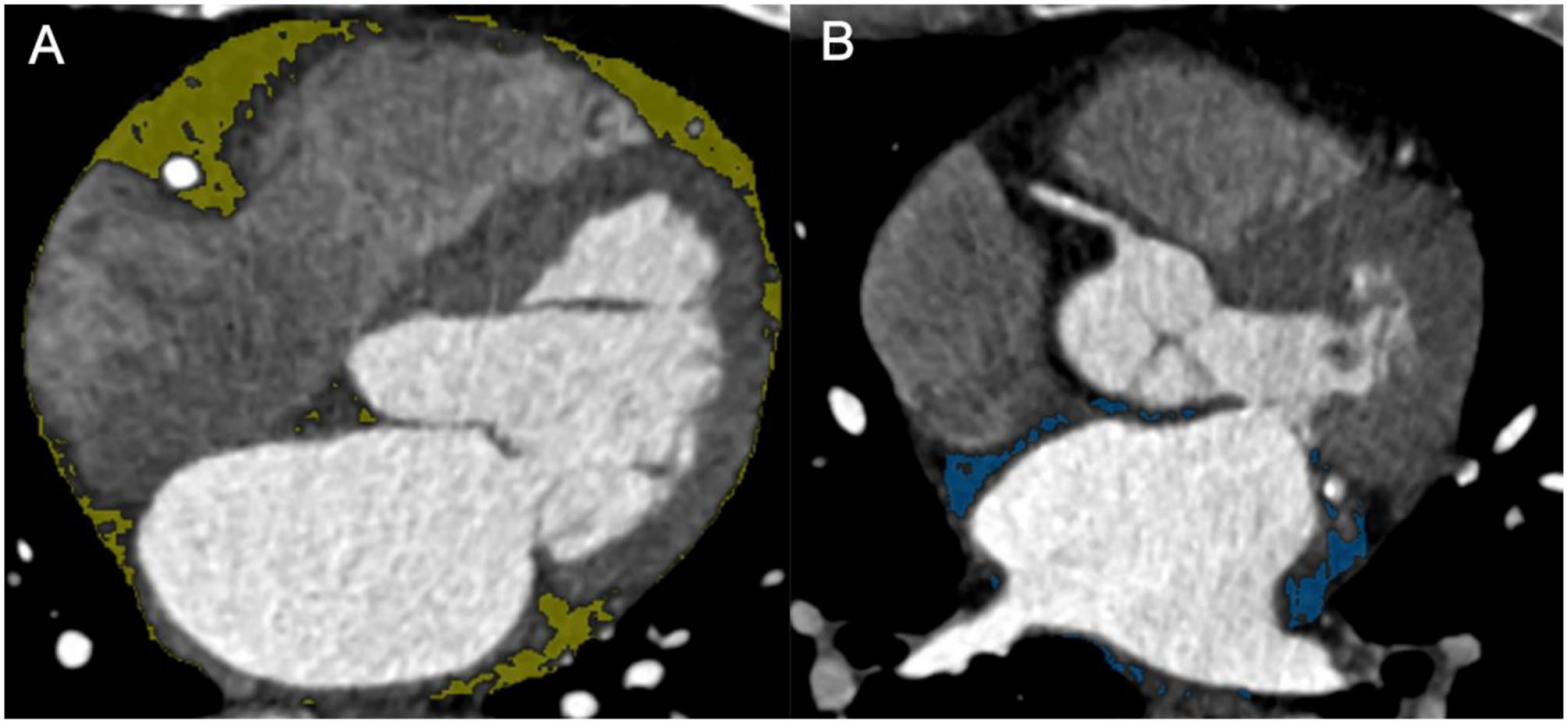
**(A)** EAT. **(B)** LA-EAT. EAT, epicardial adipose tissue; LA-EAT, epicardial adipose tissue surrounding the left atrium.

The EAT and LA-EAT were measured using the commercial post-processing workstation (SyngoVia, VB20, Siemens Healthineers, Forchheim, Germany). We adopted software (Cardiac risk analysis, SyngoVia, Research Frontier, Siemens Healthineers) to segment EAT automatically, and checked by two radiologists, who were blinded to the patients' clinical history. To delineate LA-EAT, we loaded the EAT mask into the Radiomics software (SyngoVia, Research Frontier, Siemens Healthineers) (24). Initially, the LA-EAT mask was manually tracked on the axial image, the upper boundary is the pulmonary artery, the lower boundary is the coronary sinus, and manually trimmed using EAT as a reference. Then, LA-EAT was automatically identified using the threshold of −200 to −50 HU. Meanwhile, the EAT and LA-EAT volumes in cubic centimeters were measured (Figure 3).

### Radiomic Feature Extraction

All extractions of radiomic features were put into effect by the radiomics software based on the PyRadiomics library (25) according to the Imaging Biomarker Standardization Initiative requirement (26). Six classes of radiomic features with five transformations were calculated for each volume of interest (VOI). Detailed descriptions of extracted features are shown in Supplementary Data 3.

### Feature Selection and Model Construction

Feature reproducibility was first evaluated for screening out stability features. Fifty cases were randomly selected for repeated segmentation, and Spearman's correlation coefficients were calculated for all extracted features. Features with Spearman's correlation coefficient < 0.80 were considered as having poor stability and reproducibility and were excluded for further analysis. In addition, feature dependency was evaluated by implementing mutual information calculation between all stable features and targeted labels. Features with mutual information > 0.05 were finally screened out for model establishment. Subsequently, the random forest algorithm, which was suited for a large number of heterogeneous predictors and correlated observations and selected by various radiomics studies (27), was implemented as a machine learning-based classifier in this study. Parameter estimation using a grid search with ten-fold cross-validation was applied to the training data for parameter tunings. The Gini importance of each variable can be gained by summing the Gini impurity reduction of each variable of all trees in the random forest model. The radiomics models (Rmodel) reflecting the LA-EAT features were built to differentiate the AF subtypes.

Univariate analysis was applied to assess the relevance of clinical characteristics and measured volume signatures. Variables with *p* < 0.05 were included in the following modeling. Multivariate stepwise logistic regression with a minimum Akaike information model selection criterion (28) was then implemented and screen-independent factors among the clinical model (Cmodel) and the model based on volume values (Vmodel). Additionally, a nomogram was established by integrating the radiomic features and the volume information (Commodel) to further evaluating the diagnosis value of EAT features extracted from CT images.

The same methodology and algorithm were applied to the radiomic features to explore the prognostic value for disease recurrence in patients within 1 year.

### Model Performance Assessment

This study evaluated model performance from three aspects: diagnostic accuracy, the goodness of fit, and clinical gain. The receiver operating characteristic curve (ROC), heatmap, specificity, and sensitivity were applied in training and validation cohorts for appraising the model diagnostic accuracy. The comparisons between AUC in models were performed by the Delong's test. The goodness of fit of models was appraised by the calibration curve and Brier score. The clinical net benefits of the different threshold probabilities were calculated by the decision curve analysis (DCA) in the validation cohort, thereby determining the clinical applicability of the prediction models of this study.

### Statistical Analysis

Normality was checked by the Kolmogorov-Smirnov test. The normally distributed variables are described as mean ± standard deviation, and the non-normally distributed variables are described as median (25th−75th percentiles). Use the χ^2^ test or Fisher's exact test to compare categorical variables. The Student's *t*-test and the Wilcoxon rank-sum test were used for comparing continuous variables. The statistical analysis was performed on SPSS version 25.0, R software (Version 3.6.1; Boston, MA, USA) and Python with scikit-learning package (version 0.23, https://scikit-learn.org/stable/index.html). More detailed information can be found in Supplementary Data 4. A *p*-value < 0.05 (two-sided) was considered statistically significant.

## Results

### Clinical Characteristics

The clinical characteristics and morphological parameters of the patients in the training and validation cohorts are illustrated in Table 1. No statistically significant discrepancy exists between these two cohorts except gender, which may be due to the fact that the cohort was randomly divided into the training and validation cohorts. The univariate analysis of the clinical characteristics and morphological parameters of the paroxysmal and persistent AF is summarized in Table 2. Moreover, the univariate analysis of clinical characteristics and morphological parameters of the AF recurrence is summarized in Table 3.

**Table 1:** Clinical characteristics and morphological parameters in the training and validation cohorts.

**Characteristic**		**Training cohort** **(251 patients)**	**Validation cohort** **(63 patients)**	***p***
Age (years)		62.00 (55.00, 67.00)	64.00 (57.00, 68.00)	0.13
BMI(Kg/m^2^)		25.01 ± 3.00	24.64 ± 3.48	0.41
TC (mmol/L)		4.43 ± 0.99	4.37 ± 1.16	0.67
LDL-C(mmol/L)		2.63 ± 0.84	2.60 ± 0.91	0.87
HDL-C(mmol/L)		1.22 ± 0.25	1.19 ± 0.26	0.44
TG (mmol/L)		1.58 ± 0.87	1.44 ± 0.76	0.24
Gender	Male	148 (59.0%)	46 (73.0%)	0.04
	Female	103 (41.0%)	17 (27.0%)	
Diabetes	Absent	216 (86.1%)	57 (90.5%)	0.35
	Presence	35 (13.9%)	6 (9.5%)	
Hyperlipidemia	Absent	147 (58.6%)	34 (54.0%)	0.51
	Presence	104 (41.4%)	29 (46.0%)	
Hypertension	Absent	115 (45.8%)	36 (57.1%)	0.11
	Presence	136 (54.2%)	27 (42.9%)	
LAV (mL)		114.86 ± 36.45	121.05 ± 36.95	0.24
EATV (mL)		113.02 ± 48.11	119.92 ± 60.03	0.34
LA-EATV (mL)		24.50 ± 12.63	27.21 ± 15.35	0.15

*A p-value < 0.05 was considered as statistically significant. BMI, body mass index; EATV, EAT volume; HDL-C, high-density lipoprotein cholesterol; LDL-C, low-density lipoprotein cholesterol; LAV, LA volume; LA-EATV, LA-EAT volume; TC, total cholesterol; TG, triglyceride*.

**Table 2:** Univariate analysis of clinical characteristics and morphological parameters in the PAF and PeAF training cohorts.

**Characteristic**		**PAF** **(173 patients)**	**PeAF** **(78 patients)**	***P***
Age (years)		63.00 (56.00, 68.00)	60.00 (54.00, 66.00)	0.06
BMI (Kg/m^2^)		24.51 ± 2.96	26.11 ± 2.81	<0.05
TC (mmol/L)		4.39 ± 0.99	4.53 ± 0.97	0.28
LDL-C (mmol/L)		2.57 ± 0.85	2.74 ± 0.81	0.15
HDL-C (mmol/L)		1.24 ± 0.25	1.17 ± 0.24	0.58
TG (mmol/L)		1.51 ± 0.84	1.74 ± 0.92	0.05
Gender	Male	87 (50.3%)	61 (78.2%)	<0.05
	Female	86 (49.7%)	17 (21.8%)	
Diabetes	Absent	145 (83.8%)	71 (91.0%)	0.13
	Presence	28 (16.2%)	7 (9.0%)	
Hyperlipidemia	Absent	105 (60.7%)	42 (53.8%)	0.31
	Presence	68 (39.3%)	36 (46.2%)	
Hypertension	Absent	75 (43.4%)	40 (51.3%)	0.24
	Presence	98 (56.6%)	38 (48.7%)	
LAV (mL)		100.07 ± 25.92	147.67 ± 35.08	<0.05
EATV (mL)		102.76 ± 43.69	135.76 ± 49.92	<0.05
LA-EATV (mL)		21.17 ± 10.35	31.88 ± 14.08	<0.05

*A p-value < 0.05 indicates a statistical difference. PAF, paroxysmal atrial fibrillation; PeAF, persistent atrial fibrillation; BMI, body mass index; TC, total cholesterol; HDL-C, high-density lipoprotein cholesterol; LDL-C, low-density lipoprotein cholesterol; TG, triglyceride; LAV, LA volume; EATV, EAT volume; LA-EATV, LA-EAT volume*.

**Table 3:** Univariate analysis of clinical characteristics and morphological parameters in AF non-recurrence and recurrence training cohorts.

**Characteristic**		**Non-recurrence** **(183 patients)**	**Recurrence** **(68 patients)**	***P***
Age (years)		62.00 (56.00, 67.00)	62.00 (52.25, 67.00)	0.10
BMI (Kg/m^2^)		24.98 ± 2.96	25.08 ± 3.12	0.83
TC (mmol/L)		4.41 ± 0.97	4.49 ± 1.04	0.55
LDL-C (mmol/L)		2.61 ± 0.83	2.68 ± 0.86	0.53
HDL-C (mmol/L)		1.23 ± 0.25	1.18 ± 0.26	0.19
TG (mmol/L)		1.56 ± 0.82	1.63 ± 1.00	0.56
Gender	Male	110 (60.1%)	38 (55.9%)	0.55
	Female	73 (39.9%)	30 (44.1%)	
Diabetes	Absent	158 (86.3%)	58 (85.3%)	0.83
	Presence	25 (13.7%)	10 (14.7%)	
Hyperlipidemia	Absent	108 (59.0%)	39 (57.4%)	0.81
	Presence	75 (41.0%)	29 (42.6%)	
Hypertension	Absent	80 (43.7%)	35 (51.5%)	0.27
	Presence	103 (56.3%)	33 (48.5%)	
LAV (mL)		114.34 ± 36.12	116.27 ± 37.54	0.71
EATV (mL)		113.68 ± 48.88	111.22 ± 46.31	0.72
LA-EATV (mL)		24.44 ± 12.45	24.64 ± 13.18	0.91

*A p-value < 0.05 indicates a statistical difference. PAF, paroxysmal atrial fibrillation; PeAF, persistent atrial fibrillation; BMI, body mass index; TC, total cholesterol; HDL-C, high-density lipoprotein cholesterol; LDL-C, low-density lipoprotein cholesterol; TG, triglyceride; LAV, LA volume; EATV, EAT volume; LA-EATV, LA-EAT volume*.

### Feature Selection and Radiomics Signature Construction for Distinguishing AF Types

Extracted from the VOI with nine filtering types and eight wavelet transformations were 1,674 radiomic features including 18 and 75 first-order and texture features, respectively. Ninety low reproducible and unstable features that had a correlation coefficient < 0.8 (0.7–0.8) were excluded. Afterward, 302 features were further screened out by the dependency test with mutual information > 0.05. Finally, 14 features were ranked among the remaining features for all training cohorts (Supplementary Figure 1) including four and ten first-order and textual features, respectively (Supplementary Table 1). The heatmap of selected features can be found in Supplementary Figure 2. The Rmodel based on these extracted 14 radiomic features was then built to differentiate the AF subtype.

### Development, Performance, and Validation of AF Types Prediction Models

**Figure 4:**
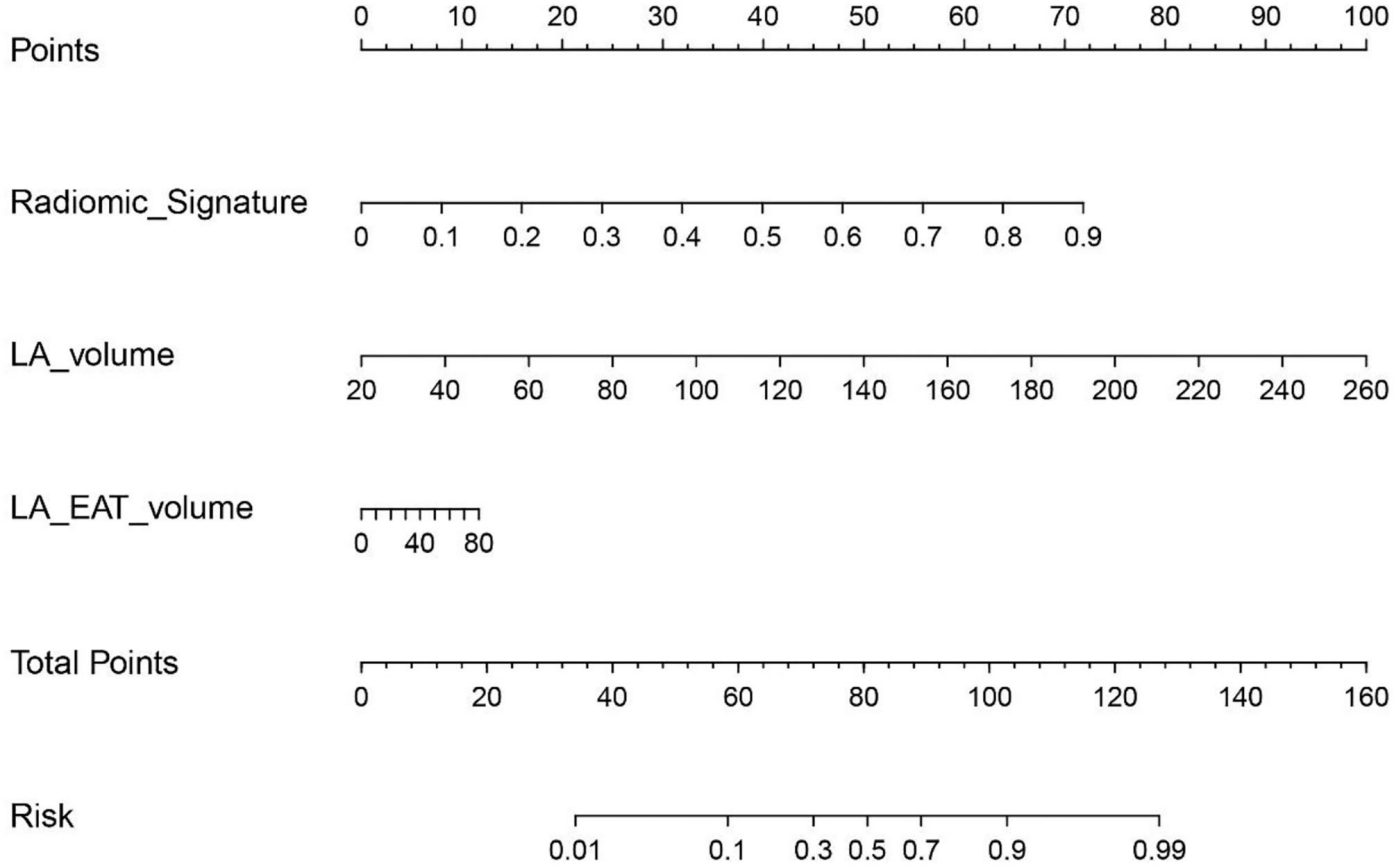
The radiomics nomogram of a personalized predictive model.

Two clinical features (including gender and BMI) and three-volume values (including LA volume, EAT volume, and LA-EAT volume) among all the clinical characteristics and volume values were selected by univariate analysis (Table 2), the Cmodel was then built based on two independent predictors (gender and BMI) of the AF subtype, and the Vmodel was built based on three independent predictors (LA, EAT, and LA-EAT volumes) of the AF subtype. Then, LA and LA-EAT volumes were selected into the radiomics signature of the Commodel by multivariate stepwise logistic regression method (Table 4). The nomogram integrating the radiomic features and the volume information is shown in Figure 4.

**Table 4:** Risk factors for AF subtype.

**Variable**	**Odds ratio**	***P*-value**
**Cmodel**		
Gender	0.30 (0.16, 0.56)	<0.001
BMI (Kg/m^2^)	1.19 (1.08, 1.32)	<0.001
**Vmodel**		
LAV (mL)	1.05 (1.04, 1.07)	<0.001
EATV (mL)	0.99 (0.97, 1.00)	0.16
LA-EATV (mL)	1.09 (1.02, 1.17)	0.02

*A p-value < 0.05 indicates a statistical difference. BMI, body mass index; LAV, LA volume; EATV, EAT volume; LA-EATV, LA-EAT volume*.

**Figure 5:**
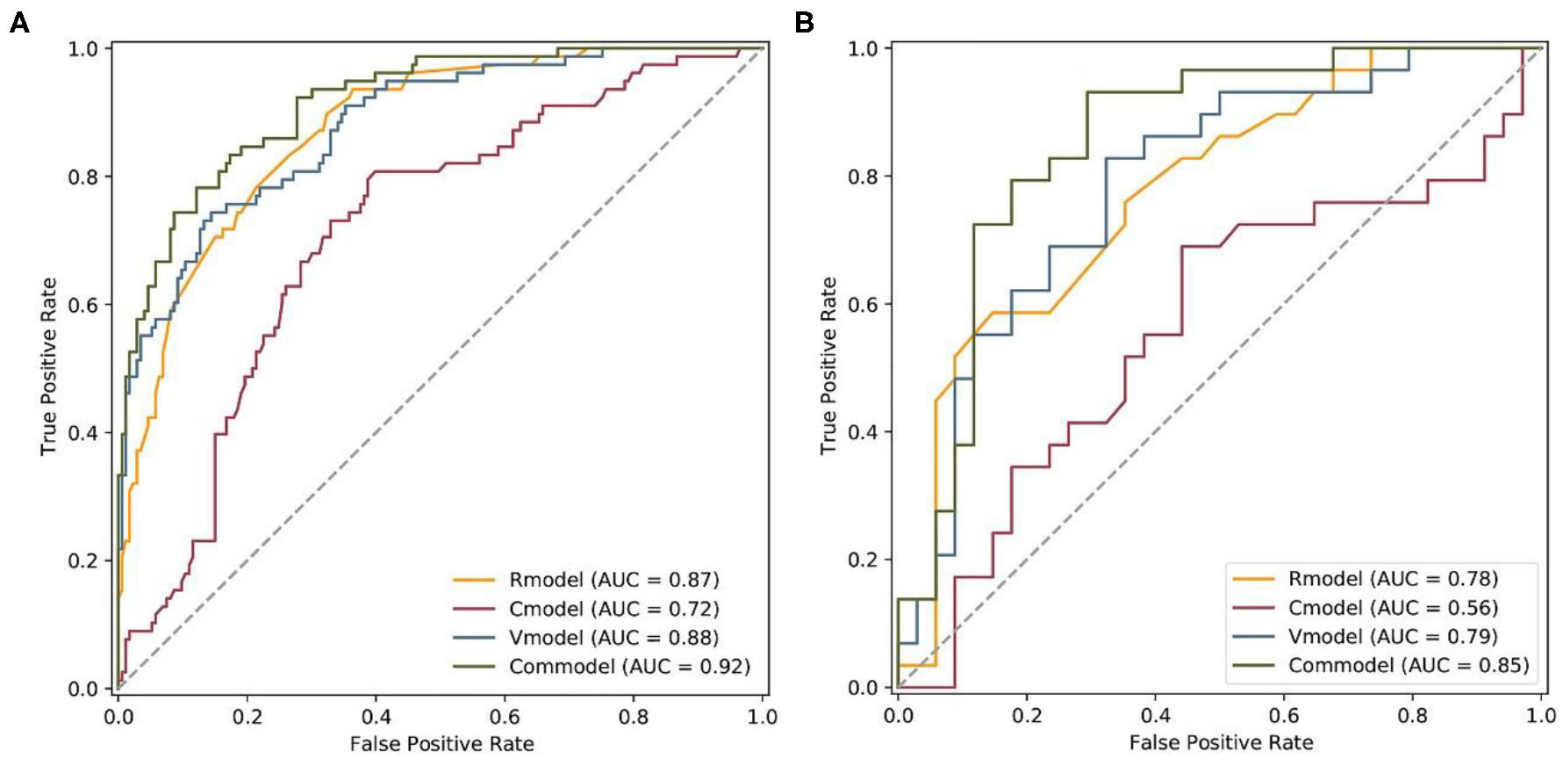
Comparison of ROCs between the Rmodel, Cmodel, Vmodel, and Commodel for the prediction of AF subtype in the **(A)** training and **(B)** validation cohorts. *Rmodel*, radiomics signature model; *Cmodel*, clinical model; *Vmodel*, the model based on volume values; *Commodel*, the model combined with both radiomic features and volume information.

The performance of all four models for differentiating the AF subtype was analyzed by ROC curve (Figures 5A,B; Table 5). In the training cohort, the Commodel demonstrated the best discrimination between PAF and PeAF with an area under the curve (AUC) of 0.915 [95% confidence interval (CI), 0.880 and 0.951], which was significantly higher than that of the Cmodel (AUC, 0.716; 95% CI, 0.649 and 0.783; *p* < 0.001), Rmodel (AUC, 0.874; 95% CI, 0.830 and 0.918; *p* = 0.01), and the Vmodel (AUC, 0.879; 95% CI, 0.835 and 0.924; *p* = 0.004). In the validation cohort, the Commodel also yielded a higher AUC (0.853; 95% CI, 0.755 and 0.951), proving a better predictive effectiveness than the Cmodel (AUC, 0.565; 95% CI, 0.417 and 0.713; *p* < 0.001),but no statistical significance was found between the Commodel and Vmodel (AUC, 0.789; 95% CI, 0.675 and 0.903; *p* = 0.14) and between the Commodel and Rmodel (AUC, 0.775; 95% CI, 0.659 and 0.892; *p* = 0.11).

**Table 5:** The performance of all the four models for differentiating the AF subtype.

**Model**		**Rmodel**	**Cmodel**	**Vmodel**	**Commodel**
Accuracy	Training cohort	0.745 (0.686, 0.798)	0.665 (0.603, 0.723)	0.821 (0.768, 0.866)	0.849 (0.798, 0.891)
	Validation cohort	0.667 (0.537, 0.781)	0.603 (0.472, 0.724)	0.730 (0.604, 0.834)	0.810 (0.691, 0.898)
Sensitivity	Training cohort	0.897 (0.808, 0.955)	0.808 (0.703, 0.888)	0.744 (0.632, 0.836)	0.782 (0.674, 0.868)
	Validation cohort	0.828 (0.642, 0.942)	0.655 (0.457, 0.821)	0.690 (0.492, 0.847)	0.793 (0.603, 0.920)
Specificity	Training cohort	0.676 (0.601, 0.745)	0.601 (0.524, 0.675)	0.856 (0.794, 0.904)	0.879 (0.820, 0.923)
	Validation cohort	0.529 (0.351, 0.702)	0.559 (0.379, 0.728)	0.765 (0.588, 0.893)	0.824 (0.655, 0.932)

*Cmodel, clinical model; Commodel, the model combined with both radiomic features and volume information; Rmodel, radiomics signature model; Vmodel, the model based on volume values*.

**Figure 6:**
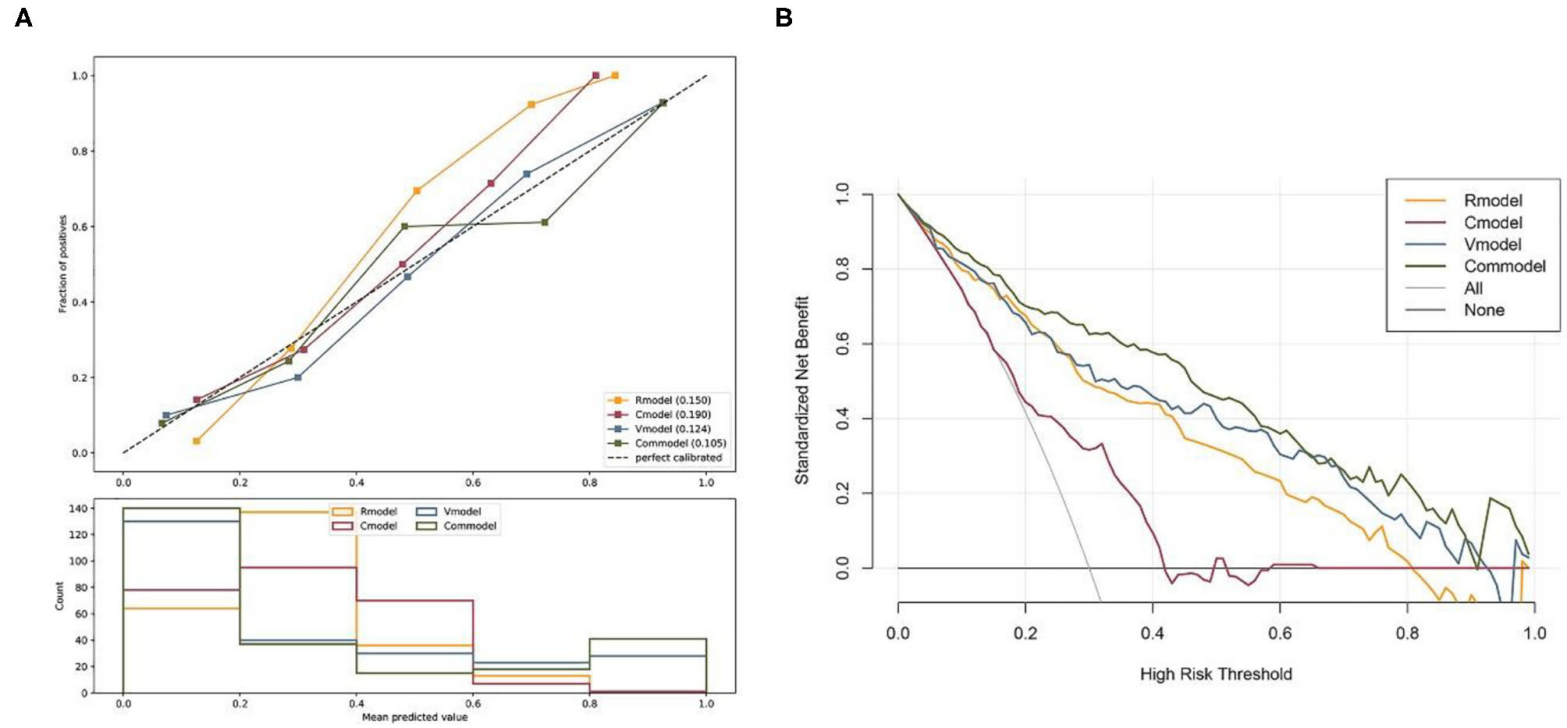
**(A)** Calibration curves of the Rmodel, Cmodel, Vmodel, and Commodel. **(B)** The decision curve analysis for the Rmodel, Cmodel, Vmodel, and Commodel. The decision curve analysis showed that the Commodel had the highest overall net benefit ratio compared with the Rmodel, Cmodel, and Vmodel. *Rmodel*, radiomics signature model; *Cmodel*, clinical model; *Vmodel*, the model based on volume values; *Commodel*, the model combined with both radiomic features and volume information.

The calibration curve of the prediction model manifested goodness of fit between observed and predicted AF subtypes in the training cohort. The Brier scores of the Rmodel, Cmodel, Vmodel, and Commodel were 0.150, 0.190, 0.124, and 0.105, respectively. Generally, a lower Brier score implies better model calibration and discrimination. Hence, the Commodel had better goodness of fit than the other three models (Figure 6A).

The DCA for all models was presented in Figure 6B. The Commodel had the highest overall net benefit ratio than the Rmodel, Cmodel, and Vmodel.

### Recurrence Prediction Model

Of the 314 enrolled patients, 79 patients had a recurrence of AF, while 235 patients had no recurrence of AF. Apply univariate analysis to assess the relevance of the clinical characteristics and measured volume signatures. No factors were associated with AF recurrence (Table 3). Twenty-six features were finally screened out in the radiomics model (Supplementary Figure 1) includes 2 and 24 first-order and textual features, respectively (Supplementary Table 2). The heatmap of selected features can be found in Supplementary Figure 2.

**Figure 7:**
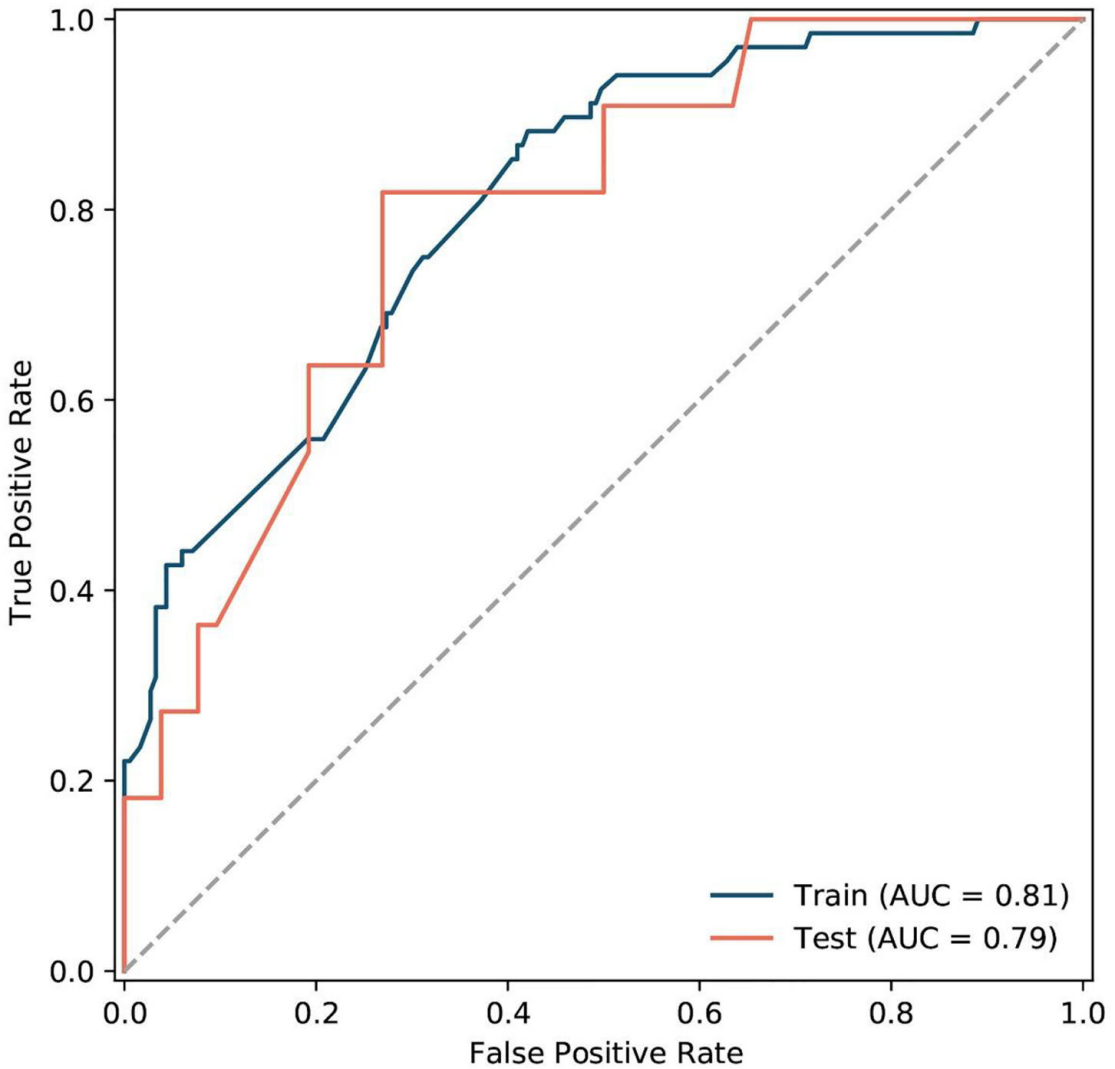
The ROC of the radiomics signatures model for predicting AF recurrence in the training and validation cohorts.

The model performance for predicting AF recurrence was analyzed by the ROC curve (Figure 7). It was performed with an AUC of 0.808 (95% CI, 0.750–0.866) and 0.793 (95% CI, 0.654–0.931) in the training and validation cohorts, respectively. Additionally, the radiomics model achieved an accuracy of 0.661 (95% CI, 0.599–0.720) and 0.635 (95% CI, 0.504–0.753), sensitivity of 0.882 (95% CI, 0.781–0.948) and 0.818 (95% CI, 0.482–0.977), and specificity of 0.579 (95% CI, 0.504–0.652) and 0.596 (95% CI, 0.451–0.730) in the training and validation cohorts.

## Discussion

This study showed that the model established by integrating the volume information and the radiomics signature of LA-EAT extracted from CT images showed the convincing differentiation ability of AF subtype. Furthermore, the performance of the Rmodel and the Vmodel were better than the Cmodel, indicating that the incorporation of CT images information into the predictive model can make up for the poor prediction in the AF subtype using the model constructed based on the clinical information alone. In addition, a quantitative radiomics prediction model based on LA-EAT features for the recurrence of AF was also preliminary developed and validated, suggesting potential in the management strategy of AF. On the contrary, no clinical and volume value model was developed due to the lack of statistical difference between the recurrent and non-recurrent groups.

This study showed that textual features accounted for the majority of the selected features (10 of 14 in the prediction of AF subtypes model; 24 of 26 in the prediction of AF recurrence model). Similarly, previous studies suggested that textual features of adipose tissue affect the histological heterogeneity of the adipose tissue. In addition, the hounsfield units of inflamed tissue is higher than that of non-inflammatory tissue (29). Indeed, inflammatory pathways resulting in structural changes within the LA were crucial in the AF mechanism. EAT is a source of inflammatory mediators (8, 30). Moreover, biopsies in AF patients have found inflammatory cells in the atrial tissue. A local inflammatory response may cause fibrosis in the surrounding atrial myocardium, and then lead to the development of AF (31). Mazurek et al. found that inflammatory activity of EAT value on fluorodeoxyglucose positron emission tomography images was strongly correlated with AF, which was absent in the subcutaneous adipose tissues (12). These may explain the finding of Kusayama et al. (32), in which the mean LA-EAT density of PAF patients on CT images was higher than that of normal people. For the same reason, the ten and four texture and first-order signatures, respectively, extracted by radiomics methods in this study were able to reflect the textural changes and voxel intensities of LA-EAT demonstrating great potential in predicting characteristics and AF recurrence.

In addition, a preliminary exploration of the prediction of AF recurrence by radiomics signatures was conducted. The literature suggests the increase in the inflammatory environment before ablation is related to the AF recurrence after catheter ablation (33) indicating that an elevated inflammatory environment may have occurred before the recurrence. Opolski et al. (34) found that the orifice area of the right superior PV is an independent predictor of postoperative new AF. The best predictive cutoff values are 4.1 cm^2^ and 3.4 mL, respectively. The majority of textual features in the radiomics signature predicting AF recurrence describes the heterogeneity in the image. Thus, the AF recurrence after catheter ablation can be predicted.

No significant difference in other clinical parameters, including age, the prevalence of hypertension, diabetes mellitus, and dyslipidemia were found in neither subtype nor recurrence groups in this study except for the difference of BMI and sex between the PAF and PeAF groups. This suggested that clinical characteristics provided limited usefulness in predicting the subtype and AF recurrence, which may lean more on CT images. In addition, The AUC value of the Cmodel in the validation cohort (AUC: 0.565) has a significant drop relative to the training cohort (AUC: 0.716). This may be due to differences in gender distribution between training and validation cohorts caused by random grouping, which may also indicate the poor generalization of the Cmodel. Despite the difference in gender distribution between the two cohorts, the commodel showed statistically better diagnostic performance than the Cmodel in both cohorts.

This study showed that LA volume being integrated into the Commodel yielded better performance than the model based radiomic features alone. Patients with PeAF had a significantly larger LA volume than the patients with PAF, which was consistent with previous studies (35). AF can cause undesirable LA remodeling. Increased LA volume leads to a greater degree of mechanical remodeling (36). On the other hand, the EAT volume showed no statistical significance in the multivariate analysis and was not an independent predictive factor for AF subtype characterization. This may suggest that the LA-EAT volume is a more reliable factor in deciding the AF subtype compared with the EAT volume as the former was focused on LA by providing dedicated LA information.

The majority of EAT studies in patients with AF remain observational. Limited studies attempted to decide on the AF subtype and recurrence by clinical or imaging parameters. Oba et al. (37) adopted an EATV index thatwas related to the AF subtype to predict the prevalence of AF. Moreover, it has been found that the PAF and PeAF development can be predicted by their cutoff values. However, only EAT around the entire heart was analyzed in the study of Oba et al. Additionally, Ciuffo et al. (17) found that LA-EAT attenuation was an independent predictor of AF recurrence. However, only two- and four-chamber views were used which may induce the missing regions that were not covered by those two views. On the contrary, a large number of radiomic features from LA-EAT were extracted in this study. Meanwhile, the clinical characteristics and volume values of LA, LA-EAT, and EAT were analyzed as well. This study also compared the performances of these three properties-based model in AF subtype prediction and further explored the predictive value of the radiomic features in AF recurrence.

This study has several limitations. First, the segmentation of EAT was automatically obtained while LA-EAT was drawn semi-automatically which can cause inconsistency. Thus, consistency assessment was applied to examine feature stability to minimize the impact of segmentation bias. Second, this was a single-center retrospective study. Several factors would potentially influence the texture analysis and establishment of predictive models, ranging from image acquisition to texture analysis. Therefore, further studies with more samples and on different scanners with different scanning protocols need to be performed to verify the reliability and reproducibility of this predictive model.

## Conclusions

To sum up, this study established a combination model integrating the radiomics signature and volume features derived from cardiac CTA that allows the pre-evaluation of AF characteristics. Furthermore, the radiomic features were also proven reliable and has the potential in predicting AF recurrence. Thus, radiomics may be a promising technology in personalizing therapy and risk management for patients with AF by characterizing the LA-EAT from the CT imaging.

## Data Availability Statement

The raw data supporting the conclusions of this article will be made available by the authors, without undue reservation.

## Author Contributions

MY, QC, ZX, YG, and SL: material preparation and data collection and analysis. WY and FY: conceptualization, resources, and methodology. MY: the first draft of the manuscript. All authors commented on previous versions of the manuscript, read and approved the final manuscript, and contributed to the study conception and design.

## Funding

This work was funded by Natural Science Foundation of China under Grant: 8217070113.

## Conflict of Interest

ZX and YG were employed by Siemens Healthineers Computed Tomography Collaboration, Shanghai, China. The remaining authors declare that the research was conducted in the absence of any commercial or financial relationships that could be construed as a potential conflict of interest.

## Publisher's Note

All claims expressed in this article are solely those of the authors and do not necessarily represent those of their affiliated organizations, or those of the publisher, the editors and the reviewers. Any product that may be evaluated in this article, or claim that may be made by its manufacturer, is not guaranteed or endorsed by the publisher.
