# Supplementary material for: Development and Validation of a Machine Learning-Based Radiomics Model on Cardiac Computed Tomography of Epicardial Adipose Tissue in Predicting Characteristics and Recurrence of Atrial Fibrillation
Source: Front Cardiovasc Med. 2022 Mar 3;9:813085. doi: 10.3389/fcvm.2022.813085 (PMC8927627; doi:10.3389/fcvm.2022.813085)
Supplement: Supplementary file 1 [file Data_Sheet_1.docx]

**SUPPLEMENTAL MATERIAL**

**Methods**

**S1: Exclusion criteria**

(1) Congenital heart disease, rheumatic heart disease, or valvular heart disease; (2) recent cardiovascular events (in the last 2 months); (3) history of malignant tumors; (4) poor image quality; (5) missing data.

**S2: CT image acquisition**

All CCTA examinations were performed on a third generation dual-source CT scanner (Somatom Force, Siemens Heathineers, Forchheim, Germany). When the CT attenuation of the region of interest (ROI) placed in the LA reaches 100HU (Hounsfield unit) for 10 seconds, the cardiac CTA was triggered, and then 50-80 mL of contrast agent (Ultravist 370, Bayer Schering, Germany) was injected at the rate of 4.0-6.0 mL/s, followed by an injection of 30 mL of saline. According to the patients’ heart rhythm, the scan mode including prospective, retrospective or high-pitch prospective modes was selected. The image parameters were as follows: tube current and tube voltage automatically determined by CARE Dose4D and CARE Kv technique; detector collimation 2× 68× 0.6 for single energy acquisition; gantry rotation 0.25 s; pitch 0.15-0.25; 512× 512 pixel matrix size. Images were reconstructed at the kernel of Bv40, with the image thickness of 0.75 mm and increment of 0.4 mm. The phase with best image quality was picked for imaging measurements and analysis.

**S3: Radiomic feature extraction methodology**

In our study, a total of 93 three-dimensional radiomic features, including 18 first-order statistics and 75 statistics-based textural features, were generated from each original CT image. Feature extraction was performed using an in-house software written in Python (Pyradiomics; version: stable; <http://www.radiomics.io/pyradiomics.html>).

1. **First-order statistics**: describe the histogram of voxel intensity values within the ROI through

commonly used and basic metrics.

**Let**: X be a set of N_p_ voxels included in the ROI,

P(i) be the first order histogram with N_g_ discrete intensity levels, where N_g_ is the number of nonzero bins, equally spaced from 0 with a width defined in the binWidth parameter.

p(i) be the normalized first order histogram and equal to $\frac{P(i)}{Np}$

1. Energy.

energy = $\sum_{i=1}^{N_{p}} (X(i)+c)^{2}$

1. Total energy.

total energy =

1. Entropy.

entropy = $-\sum_{i=1}^{N_{g}} p(i)\log_{2}$(p(i)+ϵ)

Here, ϵ is an arbitrarily small positive number (≈2.2 × 10^-16^).

1. Minimum.

Minimum = min(X)

1. 10th percentile

The 10^th^ percentile of X

1. 90th percentile

The 90^th^ percentile of X

1. Maximum.

Maximum = max(X)

The maximum gray level intensity within the ROI.

1. Mean.

mean = $\frac{1}{N_{p}}\sum_{i=1}^{N_{p}} X(i)$

The average gray level intensity within the ROI.

1. Median.

The median gray level intensity within the ROI.

1. Interquartile Range.

interquartile range = P_75_ – P_25_

Here P_25_ and P_75_ are the 25th and 75th percentile of the image array, respectively.

1. Range.

range = max(X) – min(X )

The range of gray values in the ROI.

1. Mean Absolute Deviation(MAD).

MAD = $\frac{1}{N_{p}}\sum_{i=1}^{N_{p}} \text{|}X(i)-\bar{\text{X}}\text{|}$

MAD is the mean distance of all intensity values from the Mean Value of the image array.

Where$\bar{X}$is the mean of X

1. Robust Mean Absolute Deviation(rMAD).

rMAD =

Robust Mean Absolute Deviation is the mean distance of all intensity values from the Mean Value calculated on the subset of image array with gray levels in between, or equal to the 10^th^ and 90^th^ percentile.

1. Root Mean Squared(RMS).

RMS = $\sqrt{\frac{1}{N_{p}}\sum_{i=1}^{N_{p}} (X(i)+c)^{2}}$

Here, c is optional value, defined by voxelArrayShift, which shifts the intensities to prevent negative values in X. This ensures that voxels with the lowest gray values contribute the least to RMS, instead of voxels with gray level intensity closest to 0.

1. Skewness.

Skewness = $\frac{\frac{1}{N_{p}}\sum_{i=1}^{N_{p}} (X(i)-\bar{X})^{3}}{\left[\sqrt{\frac{1}{N_{p}}\sum_{i=1}^{N_{p}} (X(i)-\bar{X})^{2}}\right]^{3}}$

Skewness measures the asymmetry of the distribution of values about the Mean value.

where$\bar{X}$is the mean of X.

1. Kurtosis.

Kurtosis = $\frac{\frac{1}{N_{p}}\sum_{i=1}^{N_{p}} (X(i)-\bar{X})^{4}}{\left[\frac{1}{N_{p}}\sum_{i=1}^{N_{p}} (X(i)-\bar{X})^{2}\right]^{2}}$

where$\bar{X}$is the mean of X.

1. Variance.

variance = $\frac{1}{N_{p}}\sum_{i=1}^{N_{p}} (X(i)-\bar{X})^{2}$

1. Uniformity.

Uniformity = $\sum_{i=1}^{N_{g}} p(i)^{2}$

1. **Statistics-based textural features** describe patterns or the spatial distribution of voxel intensities, which were calculated from respectively gray level co-occurrence matrix (GLCM), Gray Level Dependence Matrix (GLDM), gray level run length matrix (GLRLM), gray-level size zone matrix (GLSZM), and Neighbouring Gray Tone Difference Matrix (NGTDM) features.

**Gray Level Co-Occurrence Matrix (GLCM) Features**

A Gray Level Co-occurrence Matrix (GLCM) of size N_g_×N_g_ describes the second-order joint probability function of an image region constrained by the mask and is defined as P(i,j|δ,θ). The (i,j)^th^ element of this matrix represents the number of times the combination of levels i and j occur in two pixels in the image, that are separated by a distance of δ pixels along angle θ. The distance δ from the center voxel is defined as the distance according to the infinity norm. For δ = 1, this results in 2 neighbors for each of 13 angles in 3D (26-connectivity).

**Let**: ϵ be an arbitrarily small positive number (≈2.2×10^−16^)

P(i , j) be the co-occurence matrix for an arbitrary δ and θ

P(i , j) be the normalized co-occurence matrix and equal to $\frac{P(i,j)}{\sum P(i,j)}$

N_g_ be the number of discrete intensity levels in the image

p_x_(i) = $\sum_{j=1}^{N_{g}} P(i,j)$ be the marginal row probabilities

p_y_(j) = $\sum_{i=1}^{N_{g}} P(i,j)$ be the marginal column probabilities

μ_x_ be the mean gray level intensity of p_x_ and defined as μ_x_ = $\sum_{i=1}^{N_{g}} p_{x}(i)i$

μ_y_ be the mean gray level intensity of p_y_ and defined as μ_y =_ $\sum_{j=1}^{N_{g}} p_{y}(j)j$

$\sigma_{x}$be the standard deviation of$p_{x}$

$\sigma_{y}$be the standard deviation of$p_{y}$

P_x+y_(k) = $\sum_{i=1}^{N_{g}} \sum_{j=1}^{N_{g}} p(i,j)$, where i + j = k, and k = 2,3, … ,2N_g_

P_x-y_(k) = $\sum_{i=1}^{N_{g}} \sum_{j=1}^{N_{g}} p(i,j)$, where| i – j| = k, and k = 0,1, … ,N_g_ -1

HX = $\text{-}\sum_{i=1}^{N_{g}} p_{x}(i)\log_{2}(p_{x}(i)+$ϵ) be the entropy of p_x_

HY = $\text{-}\sum_{j=1}^{N_{g}} p_{y}(j)\log_{2}(p_{y}(j)+$ϵ) be the entropy of p_y_

HXY = $\text{-}\sum_{i=1}^{N_{g}} \sum_{j=1}^{N_{g}} p(i,j)\log_{2}(p(i,j)+$ϵ) be the entropy of p(i , j)

HXY1 = $\text{-}\sum_{i=1}^{N_{g}} \sum_{j=1}^{N_{g}} p(i,j)\log_{2}(p_{x}(i)p_{y}(j)+$ϵ）

HXY2 = $\text{-}\sum_{i=1}^{N_{g}} \sum_{j=1}^{N_{g}} p_{x}(i)p_{y}(j)\log_{2}(p_{x}(i)p_{y}(j)+$ϵ）

1. Autocorrelation.

autocorrelation = $\sum_{i=1}^{N_{g}} \sum_{j=1}^{N_{g}} p(i,j)ij$

1. Joint Average.

joint average = μ_x_ = $\sum_{i=1}^{N_{g}} \sum_{j=1}^{N_{g}} p(i,j)i$

1. Cluster Prominence

cluster prominence = $\sum_{i=1}^{N_{g}} \sum_{j=1}^{N_{g}} (i+j-\mu_{x}-\mu_{y})^{4}p(i,j)$

1. Cluster Shade

cluster shade = $\sum_{i=1}^{N_{g}} \sum_{j=1}^{N_{g}} (i+j-\mu_{x}-\mu_{y})^{3}p(i,j)$

1. Cluster Tendency

cluster tendency = $\sum_{i=1}^{N_{g}} \sum_{j=1}^{N_{g}} (i+j-\mu_{x}-\mu_{y})^{2}p(i,j)$

1. Contrast.

contrast = $\sum_{i=1}^{N_{g}} \sum_{j=1}^{N_{g}} (i-j)^{2}p(i,j)$

1. Correlation.

correlation = $\frac{\sum_{i=1}^{N_{g}} \sum_{j=1}^{N_{g}} p(i,j)ij-\mu_{x}\mu_{y}}{\sigma_{x}(i)\sigma_{y}(j)}$

1. Difference Average.

difference average = $\sum_{k=0}^{N_{g}-1} kp_{x-y}(k)$

1. Difference Entropy.

difference entropy = $\sum_{k=0}^{N_{g}-1} p_{x-y}(k)\log_{2}(p_{x-y}(k)+$ϵ）

1. Difference Variance.

difference variance = $\sum_{k=0}^{N_{g}-1} (k-DA)^{2}p_{x-y}(k)$

1. Joint Energy.

joint energy = $\sum_{i=1}^{N_{g}} \sum_{j=1}^{N_{g}} (p(i,j))^{2}$

1. Joint Entropy.

joint entropy = $-\sum_{i=1}^{N_{g}} \sum_{j=1}^{N_{g}} p(i,j)\log_{2}(p(i,j)+$ϵ)

1. Informational Measure of Correlation (IMC) 1.

IMC1 = $\frac{HXY-HXY1}{\max\left\{HX,HY\right\}}$

1. Informational Measure of Correlation (IMC) 2.

IMC2 = $\sqrt{1-e^{-2(HXY2-HXY)}}$

1. Inverse Difference Moment (IDM).

IDM = $\sum_{k=0}^{N_{g}-1} \frac{p_{x-y}(k)}{1+k^{2}}$

1. Maximal Correlation Coefficient (MCC).

MCC = $\sqrt{\sec ond\text{ largest eigenvalue of Q}}$

Q(i , j) = $\sum_{k=0}^{N_{g}} \frac{p(i,k)p(j,k)}{p_{x}(i)p_{y}(k)}$

1. Inverse Difference Moment Normalized (IDMN).

IDMN = $\sum_{k=0}^{N_{g}-1} \frac{p_{x-y}(k)}{1+(\frac{k^{2}}{N_{g}^{2}})}$

1. Inverse Difference (ID).

ID = $\sum_{k=0}^{N_{g}-1} \frac{p_{x-y}(k)}{1+k}$

1. Inverse Difference Normalized (IDN).

IDN = $\sum_{k=0}^{N_{g}-1} \frac{p_{x-y}(k)}{1+(\frac{k}{N_{g}})}$

1. Inverse Variance.

inverse variance = $\sum_{k=1}^{N_{g}-1} \frac{p_{x-y}(k)}{k^{2}}$

1. Maximum Probability.

maximum probability = max$(p(i,j))$

1. Sum Average.

sum average = $\sum_{k=2}^{2N_{g}} p_{x+y}(k)k$

1. Sum Entropy.

sum entropy = $\sum_{k=2}^{2N_{g}} p_{x+y}(k)\log_{2}(p_{x+y}(k)+$ϵ)

1. Sum of Squares.

sum squares = $\sum_{i=1}^{N_{g}} \sum_{j=1}^{N_{g}} (i-\mu_{x})^{2}p(i,j)$

**Gray Level Run Length Matrix (GLRLM) Features**

A Gray Level Run Length Matrix (GLRLM) quantifies gray level runs, which are defined as the length in number of pixels, of consecutive pixels that have the same gray level value. In a gray level run length matrix P(i , j |θ), the (i , j)^th^ element describes the number of runs with gray level i and length j occur in the image (ROI) along angle θ.

**Let**: N_g_ be the number of discreet intensity values in the image

N_r_ be the number of discreet run lengths in the image

N_p_ be the number of voxels in the image

N_r_(θ) be the number of runs in the image along angle θ, which is equal to $\sum_{i=1}^{N_{g}} \sum_{j=1}^{N_{r}} p(i,j\text{ | }\theta)$ and 1≤N_r_(θ)≤N_p_

P(i , j | θ) be the run length matrix for an arbitrary direction θ

p(i , j | θ) be the normalized run length matrix, defined as p(i , j | θ) = $\frac{P(i,j|\theta)}{N_{r}(\theta)}$

1. Short Run Emphasis (SRE).

SRE = $\frac{\sum_{i=1}^{N_{g}} \sum_{j=1}^{N_{r}} \frac{p(i,j|\theta)}{j^{2}}}{N_{r}(\theta)}$

1. Long Run Emphasis (LRE).

LRE = $\frac{\sum_{i=1}^{N_{g}} \sum_{j=1}^{N_{r}} p(i,j|\theta)j^{2}}{N_{r}(\theta)}$

1. Gray Level Non-Uniformity (GLN).

GLN = $\frac{\sum_{i=1}^{N_{g}} (\sum_{j=1}^{N_{r}} p(i,j|\theta))^{2}}{N_{r}(\theta)}$

1. Gray Level Non-Uniformity Normalized (GLNN).

GLNN = $\frac{\sum_{i=1}^{N_{g}} (\sum_{j=1}^{N_{r}} p(i,j|\theta))^{2}}{N_{r}(\theta)^{2}}$

1. Run Length Non-Uniformity (RLN).

RLN = $\frac{\sum_{j=1}^{N_{r}} (\sum_{i=1}^{N_{g}} p(i,j|\theta))^{2}}{N_{r}(\theta)}$

1. Run Length Non-Uniformity Normalized (RLNN).

RLNN = $\frac{\sum_{j=1}^{N_{r}} (\sum_{i=1}^{N_{g}} p(i,j|\theta))^{2}}{N_{r}(\theta)^{2}}$

1. Run Percentage (RP).

RP = $\frac{N_{r}(\theta)}{N_{p}}$

1. Gray Level Variance (GLV).

GLV = $\sum_{i=1}^{N_{g}} \sum_{j=1}^{N_{r}} p(i,j\text{|}\theta)(i-u)^{2}$

Here, μ = $\sum_{i=1}^{N_{g}} \sum_{j=1}^{N_{r}} p(i,j\text{|}\theta)i$

1. Run Variance (RV).

RV = $\sum_{i=1}^{N_{g}} \sum_{j=1}^{N_{r}} p(i,j\text{|}\theta)(j-u)^{2}$

Here, μ = $\sum_{i=1}^{N_{g}} \sum_{j=1}^{N_{r}} p(i,j\text{|}\theta)j$

1. Run Entropy (RE).

RE = $-\sum_{i=1}^{N_{g}} \sum_{j=1}^{N_{r}} p(i,j|\theta)\log_{2}(p(i,j|\theta)+$ϵ)

Here, ϵ is an arbitrarily small positive number (≈2.2×10^−16^).

1. Low Gray Level Run Emphasis (LGLRE).

LGLRE = $\frac{\sum_{i=1}^{N_{g}} \sum_{j=1}^{N_{r}} \frac{p(i,j|\theta)}{i^{2}}}{N_{r}(\theta)}$

1. High Gray Level Run Emphasis (HGLRE).

HGLRE = $\frac{\sum_{i=1}^{N_{g}} \sum_{j=1}^{N_{r}} p(i,j|\theta)i^{2}}{N_{r}(\theta)}$

1. Short Run Low Gray Level Emphasis (SRLGLE).

SRLGLE = $\frac{\sum_{i=1}^{N_{g}} \sum_{j=1}^{N_{r}} \frac{p(i,j|\theta)}{i^{2}j^{2}}}{N_{r}(\theta)}$

1. Short Run High Gray Level Emphasis (SRHGLE).

SRHGLE = $\frac{\sum_{i=1}^{N_{g}} \sum_{j=1}^{N_{r}} \frac{p(i,j|\theta)i^{2}}{j^{2}}}{N_{r}(\theta)}$

1. Long Run Low Gray Level Emphasis (LRLGLE).

LRLGLE = $\frac{\sum_{i=1}^{N_{g}} \sum_{j=1}^{N_{r}} \frac{p(i,j|\theta)j^{2}}{j^{2}}}{N_{r}(\theta)}$

1. Long Run High Gray Level Emphasis (LRHGLE).

LRHGLE = $\frac{\sum_{i=1}^{N_{g}} \sum_{j=1}^{N_{r}} p(i,j|\theta)i^{2}j^{2}}{N_{r}(\theta)}$

**Gray Level Size Zone Matrix (GLSZM) Features**

A Gray Level Size Zone (GLSZM) quantifies gray level zones in an image. A gray level zone is defined as a the number of connected voxels that share the same gray level intensity. A voxel is considered connected if the distance is 1 according to the infinity norm (26-connected region in a 3D,8-connected region in 2D). In a gray level size zone matrix P(i , j) the (i , j)^th^ element equals the number of zones with gray level i and size j appear in image. Contrary to GLCM and GLRLM, the GLSZM is rotation independent, with only one matrix calculated for all directions in the ROI.

**Let**: N_g_ be the number of discreet intensity values in the image

N_s_ be the number of discreet zone sizes in the image

N_p_ be the number of voxels in the image

N_z_ be the number of zones in the ROI, which is equal to $\sum_{i=1}^{N_{g}} \sum_{j=1}^{N_{s}} p(i,j)$ and 1≤N_z_≤N_p_

P(i , j) be the size zone matrix

p( i , j) be the normalized size zone matrix, defined as p( i , j) = $\frac{P(i,j)}{N_{z}}$

1. Small Area Emphasis (SAE).

SAE = $\frac{\sum_{i=1}^{N_{g}} \sum_{j=1}^{N_{s}} \frac{p(i,j)}{j^{2}}}{N_{z}}$

1. Large Area Emphasis (LAE).

LAE = $\frac{\sum_{i=1}^{N_{g}} \sum_{j=1}^{N_{s}} P(i,j)j^{2}}{N_{z}}$

1. Gray Level Non-Uniformity (GLN).

GLN = $\frac{\sum_{i=1}^{N_{g}} (\sum_{j=1}^{N_{s}} P(i,j))^{2}}{N_{z}}$

1. Gray Level Non-Uniformity Normalized (GLNN).

GLNN = $\frac{\sum_{i=1}^{N_{g}} (\sum_{j=1}^{N_{s}} P(i,j))^{2}}{N_{z}^{2}}$

1. **Size-Zone Non-Uniformity (SZN).**

**SZN =** $\frac{\sum_{j=1}^{N_{s}} (\sum_{i=1}^{N_{g}} P(i,j))^{2}}{N_{z}}$

1. Size-Zone Non-Uniformity Normalized (SZNN).

SZNN = $\frac{\sum_{j=1}^{N_{s}} (\sum_{i=1}^{N_{g}} P(i,j))^{2}}{N_{z}^{2}}$

1. Zone Percentage (ZP).

ZP = $\frac{N_{z}}{N_{p}}$

1. Gray Level Variance (GLV).

GLV = $\sum_{i=1}^{N_{g}} \sum_{j=1}^{N_{s}} p(i,j)(i-u)^{2}$

Here, μ = $\sum_{i=1}^{N_{g}} \sum_{j=1}^{N_{s}} p(i,j)i$

1. Zone Variance (ZV).

ZV = $\sum_{i=1}^{N_{g}} \sum_{j=1}^{N_{s}} p(i,j)(j-u)^{2}$

Here, μ = $\sum_{i=1}^{N_{g}} \sum_{j=1}^{N_{s}} p(i,j)j$

1. Zone Entropy (ZE).

ZE = $-\sum_{i=1}^{N_{g}} \sum_{j=1}^{N_{s}} p(i,j)\log_{2}(p(i,j)+$ϵ)

Here, ϵ is an arbitrarily small positive number (≈2.2×10^−16^).

1. Low Gray Level Zone Emphasis (LGLZE).

LGLZE = $\frac{\sum_{i=1}^{N_{g}} \sum_{j=1}^{N_{s}} \frac{p(i,j)}{i^{2}}}{N_{z}}$

1. High Gray Level Zone Emphasis (HGLZE).

HGLZE = $\frac{\sum_{i=1}^{N_{g}} \sum_{j=1}^{N_{s}} P(i,j)i^{2}}{N_{z}}$

1. Small Area Low Gray Level Emphasis (SALGLE).

SALGLE = $\frac{\sum_{i=1}^{N_{g}} \sum_{j=1}^{N_{s}} \frac{p(i,j)}{i^{2}j^{2}}}{N_{z}}$

1. Small Area High Gray Level Emphasis (SAHGLE).

SAHGLE = $\frac{\sum_{i=1}^{N_{g}} \sum_{j=1}^{N_{s}} \frac{p(i,j)i^{2}}{j^{2}}}{N_{z}}$

1. Large Area Low Gray Level Emphasis (LALGLE).

LALGLE = $\frac{\sum_{i=1}^{N_{g}} \sum_{j=1}^{N_{s}} \frac{p(i,j)j^{2}}{i^{2}}}{N_{z}}$

1. Large Area High Gray Level Emphasis (LAHGLE).

LAHGLE = $\frac{\sum_{i=1}^{N_{g}} \sum_{j=1}^{N_{s}} P(i,j)i^{2}j^{2}}{N_{z}}$

**Gray Level Dependence Matrix (GLDM) Features**

A Gray Level Dependence Matrix (GLDM) quantifies gray level dependencies in an image. A gray level dependency is defined as a the number of connected voxels within distance δ that are dependent on the center voxel. A neighbouring voxel with gray level j is considered dependent on center voxel with gray level i if |i-j|≤α. In a gray level dependence matrix P(i,j) the (i,j)^th^ element describes the number of times a voxel with gray level i with j dependent voxels, in its neighbourhood appears in image.

**Let**: N_g_ be the number of discreet intensity values in the image

N_d_ be the number of discreet dependency sizes in the image

N_z_ be the number of dependency zones in the image, which is equal to $\sum_{i=1}^{N_{g}} \sum_{j=1}^{N_{d}} P(i,j)$

P(i , j) be the dependence matrix

p( i , j) be the normalized dependence matrix, defined as p(i,j) = $\frac{P(i,j)}{N_{z}}$

1. Small Dependence Emphasis (SDE).

SDE = $\frac{\sum_{i=1}^{N_{g}} \sum_{j=1}^{N_{d}} \frac{p(i,j)}{i^{2}}}{N_{z}}$

1. Large Dependence Emphasis (LDE).

LDE = $\frac{\sum_{i=1}^{N_{g}} \sum_{j=1}^{N_{d}} P(i,j)j^{2}}{N_{z}}$

1. Gray Level Non-Uniformity (GLN).

GLN = $\frac{\sum_{i=1}^{N_{g}} (\sum_{j=1}^{N_{d}} P(i,j))^{2}}{N_{z}}$

1. Dependence Non-Uniformity (DN).

DN = $\frac{\sum_{j=1}^{N_{d}} (\sum_{i=1}^{N_{g}} P(i,j))^{2}}{N_{z}}$

1. Dependence Non-Uniformity Normalized (DNN).

DNN = $\frac{\sum_{j=1}^{N_{d}} (\sum_{i=1}^{N_{g}} P(i,j))^{2}}{N_{z}^{2}}$

1. Gray Level Variance (GLV).

GLV = $\sum_{i=1}^{N_{g}} \sum_{j=1}^{N_{d}} p(i,j)(i-u)^{2}$ , where μ = $\sum_{i=1}^{N_{g}} \sum_{j=1}^{N_{d}} ip(i,j)$

1. Dependence Variance (DV).

DV = $\sum_{i=1}^{N_{g}} \sum_{j=1}^{N_{d}} p(i,j)(j-u)^{2}$ , where μ = $\sum_{i=1}^{N_{g}} \sum_{j=1}^{N_{d}} jp(i,j)$

1. Dependence Entropy (DE).

DE = $-\sum_{i=1}^{N_{g}} \sum_{j=1}^{N_{d}} p(i,j)\log_{2}(p(i,j)+$ϵ)

1. Low Gray Level Emphasis (LGLE).

LGLE = $\frac{\sum_{i=1}^{N_{g}} \sum_{j=1}^{N_{d}} \frac{p(i,j)}{i^{2}}}{N_{z}}$

1. High Gray Level Emphasis (HGLE).

HGLE = $\frac{\sum_{i=1}^{N_{g}} \sum_{j=1}^{N_{d}} P(i,j)i^{2}}{N_{z}}$

1. Small Dependence Low Gray Level Emphasis (SDLGLE).

SDLGLE = $\frac{\sum_{i=1}^{N_{g}} \sum_{j=1}^{N_{d}} \frac{p(i,j)}{i^{2}j^{2}}}{N_{z}}$

1. Small Dependence High Gray Level Emphasis (SDHGLE).

Measures the joint distribution of small dependence with higher gray-level values.

1. Large Dependence Low Gray Level Emphasis (LDLGLE).

LDLGLE = $\frac{\sum_{i=1}^{N_{g}} \sum_{j=1}^{N_{d}} \frac{p(i,j)j^{2}}{i^{2}}}{N_{z}}$

1. Large Dependence High Gray Level Emphasis (LDHGLE).

LDHGLE = $\frac{\sum_{i=1}^{N_{g}} \sum_{j=1}^{N_{d}} P(i,j)i^{2}j^{2}}{N_{z}}$

**Neighbouring Gray Tone Difference Matrix (NGTDM) Features**

A Neighbouring Gray Tone Difference Matrix quantifies the difference between a gray value and the average gray value of its neighbours within distance *δ*. The sum of absolute differences for gray level *i* is stored in the matrix. Let X*gl* be a set of segmented voxels and *xgl*(*jx*,*jy*,*jz*)∈X*gl* be the gray level of a voxel at postion (*jx*,*jy*,*jz*), then the average gray level of the neigbourhood is:

$$\begin{matrix} \bar{A}_{i}=\bar{A}(j_{x},j_{y},j_{z})=\frac{1}{W}\sum_{k_{x}=-\delta}^{\delta} \sum_{k_{\text{y}}=-\delta}^{\delta} \sum_{k_{z}=-\delta}^{\delta} x_{gl}(j_{x}+k_{x},j_{y}+k_{y,}j_{z}+k_{z}) \\ \end{matrix}$$

Where $(k_{x},k_{y},k_{z})\neq(0,0,0)and\text{ }x_{gl}(j_{x}+k_{x},j_{y}+k_{y},j_{z}+k_{z})\in X_{gl}$

Here, W is the number of voxels in the neighbourhood that are also in $X_{gl}$ .

**Let**: $n_{i}$be the number of voxels in$X_{gl}$with gray level i

$N_{v,p}$be the total number of voxels in $X_{gl}$and equal to $\sum n_{i}$(i.e. the number of voxels with a valid region; at least 1 neighbor). $N_{v,p}$≤N_p_ , where N_p_ is the total number of voxels in the ROI.

$p_{i}$be the gray level probability and equal to $n_{i}/N_{v}$

$s_{i}=$ $\left\{\begin{matrix} \sum^{n_{i}} |i-\bar{A}_{i}|\text{ }for\text{ }n_{i}\neq0 \\ \text{ }0\text{ }for\text{ n}_{i}=0 \end{matrix}\right.$ be the sum of absolute differences for gray level i

$N_{g}$be the number of discreet gray levels

$N_{g,p}$be the number of gray levels where $p_{i}\neq0$

1. Coarseness.

coarseness = $\frac{1}{\sum_{i=1}^{N_{g}} p_{i}s_{i}}$

1. Contrast.

contrast =$\begin{matrix} \left(\frac{1}{N_{g,p}(N_{g,p}-1)}\sum_{i=1}^{N_{g}} \sum_{j=1}^{N_{g}} p_{i}p_{j}(i-j)^{2}\right)\left(\frac{1}{N_{v,p}}\sum_{i=1}^{N_{g}} s_{i}\right), \\ where\text{ }p_{i}\neq0,p_{j}\neq0 \end{matrix}$

1. Busyness.

busyness = $\frac{\sum_{i=1}^{N_{g}} p_{i}s_{i}}{\sum_{i=1}^{N_{g}} \sum_{j=1}^{N_{g}} |ip_{i}-jp_{j}|}\text{，}where\text{ }p_{i}\neq0,p_{j}\neq0$

1. Complexity.

Complexity = $\frac{1}{N_{v,p}}\sum_{i=1}^{N_{g}} \sum_{j=1}^{N_{g}} |i-j|\frac{p_{i}s_{i}+p_{j}s_{j}}{p_{i}+p_{j}},where\text{ }p_{i}\neq0,p_{j}\neq0$

1. Strength.

strength = $\frac{\sum_{i=1}^{N_{g}} \sum_{j=1}^{N_{g}} (p_{i}+p_{j})(i-j)^{2}}{\sum_{i=1}^{N_{g}} s_{i}},where\text{ }p_{i}\neq0,p_{j}\neq0$

**The detailed composition of radiomic features extracted**

**
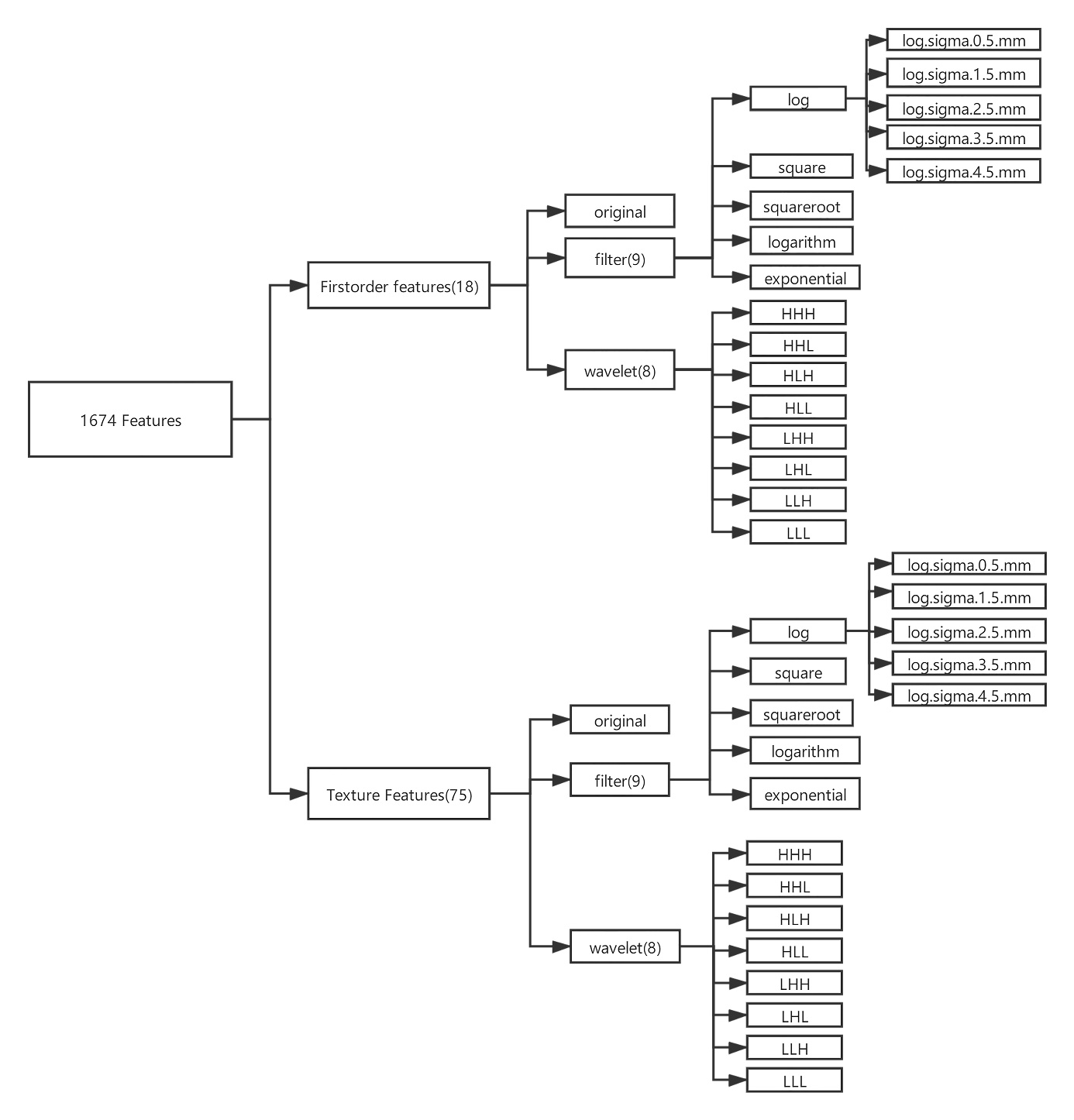
**

**S4: Statistical analysis**

Random forest classification was performed using “RandomForestClassifier” function in scikit-learn package. The multivariate logistic regression was obtained by using the rms package. The calibration curve and Brier score was obtained by scikit-learning package. The DCA was calculated by the rmda package.

**Figure S1: feature importance based on Gini index**

**
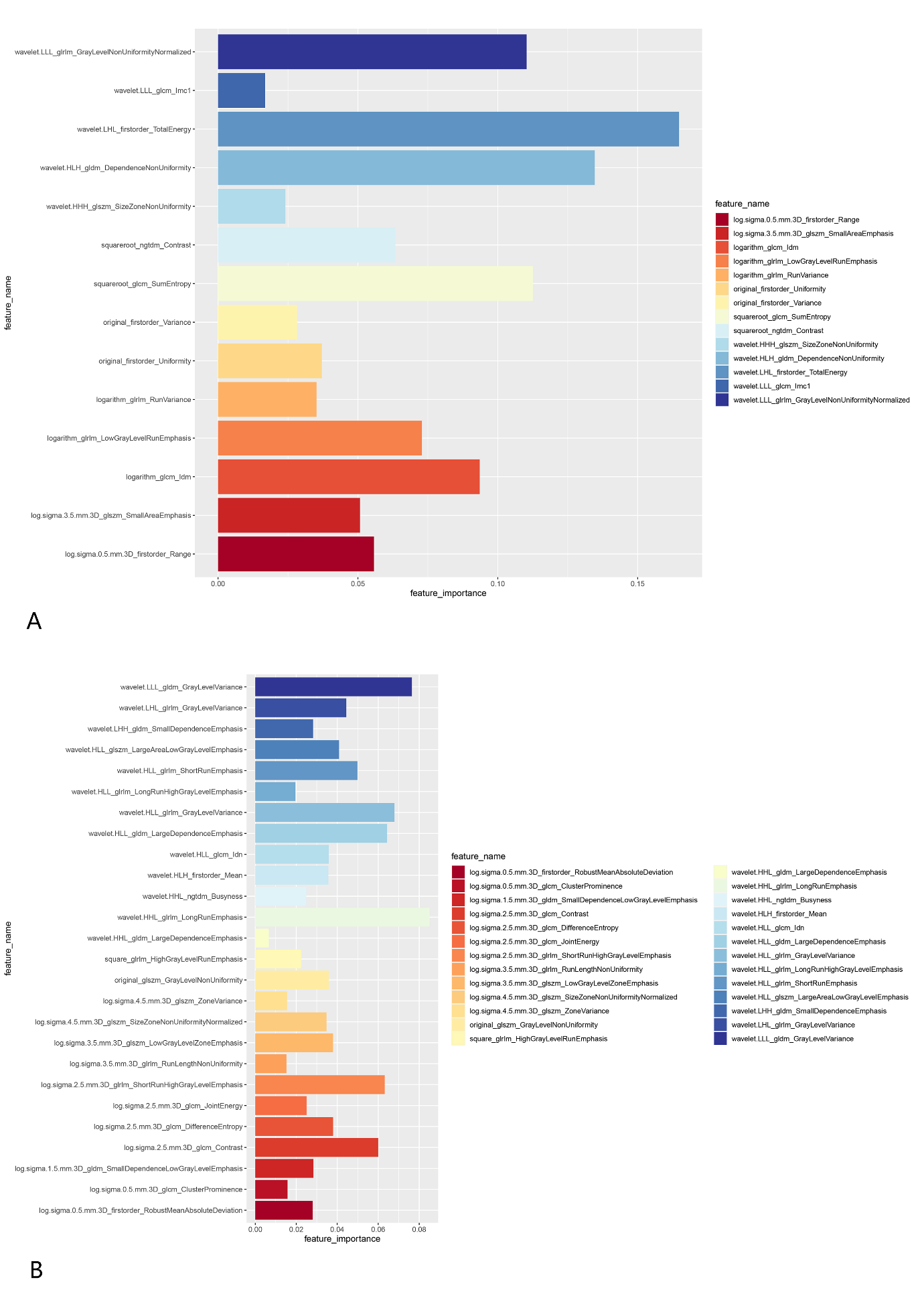
**

X axis represents the Gini-importance score and Y axis is the name of features. (A). fourteen features with Top Gini Importance Values for differentiating the subtype of AF. (B). Twenty-six features with Top Gini Importance Values for predict AF recurrence.

**Figure S2: heatmap of selected features**

**
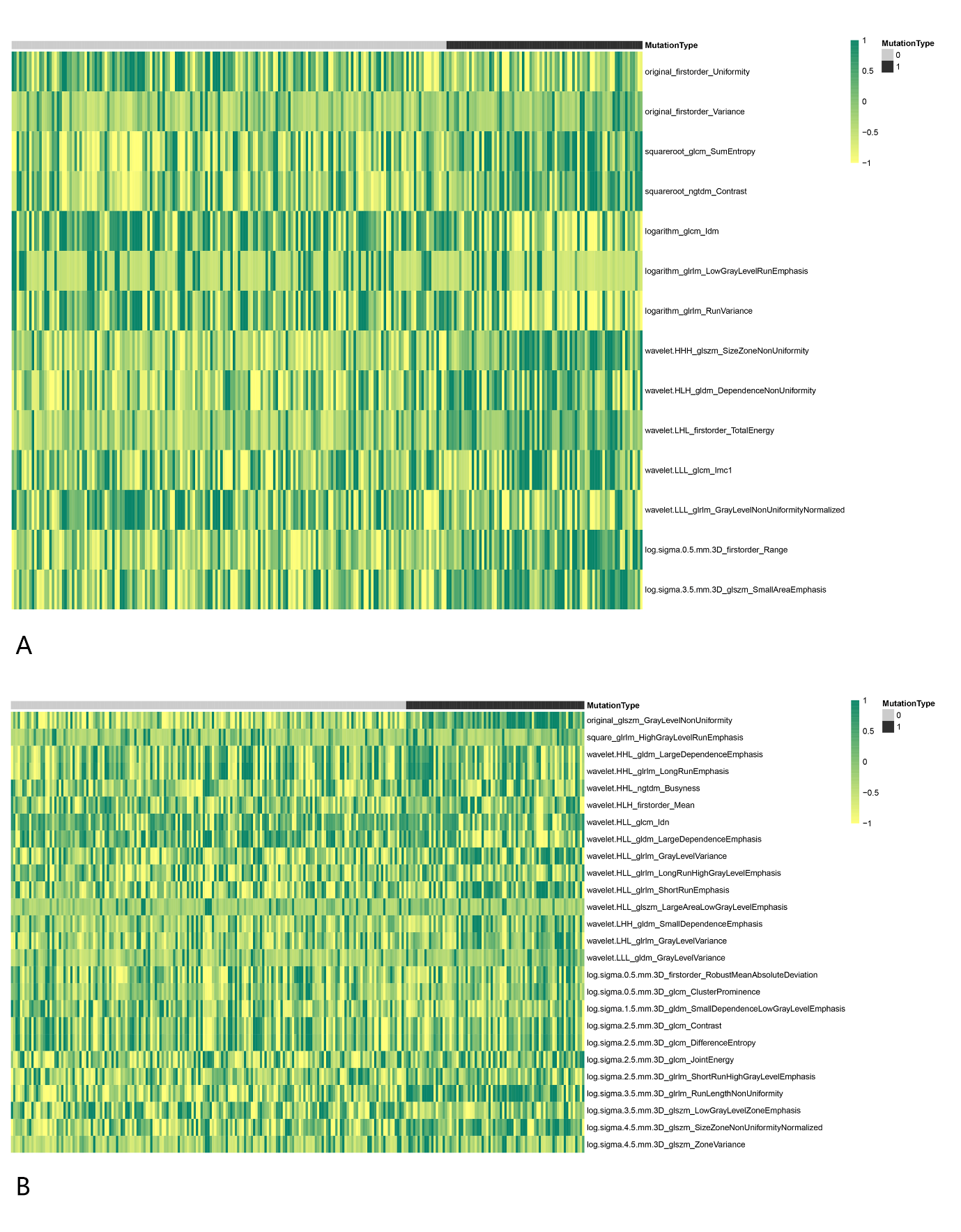
**

radiomics features had a reasonably good discrimination ability of (A)subtype of AF and (B)AF recurrence.

A: 0, PAF ; 1,PeAF. B: 0, non-recurrence; 1, recurrence. PAF, paroxysmal atrial fibrillation; PeAF, persistent atrial fibrillation.

**Table S1: Selected 14 radiomics features for prediction of subtype of AF**

| **Features** | **Filter_type** | **Wavelet** | **MI_value** | **r_spearman** |
| --- | --- | --- | --- | --- |
| original_firstorder_Uniformity |  |  | 0.06 | 0.98 |
| original_firstorder_Variance |  |  | 0.07 | 0.98 |
| squareroot_glcm_SumEntropy | square root |  | 0.08 | 0.99 |
| squareroot_ngtdm_Contrast | square root |  | 0.08 | 0.92 |
| logarithm_glcm_Idm | logarithm |  | 0.11 | 0.99 |
| logarithm_glrlm_LowGrayLevelRunEmphasis | logarithm |  | 0.08 | 0.96 |
| logarithm_glrlm_RunVariance | logarithm |  | 0.07 | 0.99 |
| wavelet.HHH_glszm_SizeZoneNonUniformity | wavelet | HHH | 0.09 | 1.00 |
| wavelet.HLH_gldm_DependenceNonUniformity | wavelet | HLH | 0.06 | 1.00 |
| wavelet.LHL_firstorder_TotalEnergy | wavelet | LHL | 0.11 | 0.99 |
| wavelet.LLL_glcm_Imc1 | wavelet | LLL | 0.05 | 1.00 |
| wavelet.LLL_glrlm_GrayLevelNonUniformityNormalized | wavelet | LLL | 0.07 | 1.00 |
| log.sigma.0.5.mm.3D_firstorder_Range | log |  | 0.12 | 1.00 |
| log.sigma.3.5.mm.3D_glszm_SmallAreaEmphasis | log |  | 0.05 | 0.98 |

glcm, gray level co-occurrence matrix; ngtdm, neighbouring gray tone difference matrix; gldm, Gray Level Dependence Matrix; glrlm, gray level run length matrix; glszm, gray level size zone matrix; Imc1, Informational measure of correlation 1; Idm, Inverse Difference Moment.

**Table S2: Selected 26 radiomics features for prediction of AF recurrence**

| **Features** | **Filter_type** | **Wavelet** | **MI_value** | **r_spearman** |
| --- | --- | --- | --- | --- |
| original_glszm_GrayLevelNonUniformity |  |  | 0.06 | 0.99 |
| square_glrlm_HighGrayLevelRunEmphasis | square |  | 0.05 | 0.94 |
| wavelet.HHL_gldm_LargeDependenceEmphasis | wavelet | HHL | 0.06 | 1.00 |
| wavelet.HHL_glrlm_LongRunEmphasis | wavelet | HHL | 0.06 | 1.00 |
| wavelet.HHL_ngtdm_Busyness | wavelet | HHL | 0.08 | 0.99 |
| wavelet.HLH_firstorder_Mean | wavelet | HLH | 0.06 | 0.99 |
| wavelet.HLL_glcm_Idn | wavelet | HLL | 0.08 | 0.93 |
| wavelet.HLL_gldm_LargeDependenceEmphasis | wavelet | HLL | 0.06 | 1.00 |
| wavelet.HLL_glrlm_GrayLevelVariance | wavelet | HLL | 0.05 | 0.99 |
| wavelet.HLL_glrlm_LongRunHighGrayLevelEmphasis | wavelet | HLL | 0.05 | 0.93 |
| wavelet.HLL_glrlm_ShortRunEmphasis | wavelet | HLL | 0.05 | 1.00 |
| wavelet.HLL_glszm_LargeAreaLowGrayLevelEmphasis | wavelet | HLL | 0.06 | 0.99 |
| wavelet.LHH_gldm_SmallDependenceEmphasis | wavelet | LHH | 0.07 | 0.99 |
| wavelet.LHL_glrlm_GrayLevelVariance | wavelet | LHL | 0.06 | 0.97 |
| wavelet.LLL_gldm_GrayLevelVariance | wavelet | LLL | 0.05 | 0.99 |
| log.sigma.0.5.mm.3D_firstorder_RobustMeanAbsoluteDeviation | log |  | 0.09 | 1.00 |
| log.sigma.0.5.mm.3D_glcm_ClusterProminence | log |  | 0.06 | 1.00 |
| log.sigma.1.5.mm.3D_gldm_SmallDependenceLowGrayLevelEmphasis | log |  | 0.08 | 0.98 |
| log.sigma.2.5.mm.3D_glcm_Contrast | log |  | 0.07 | 1.00 |
| log.sigma.2.5.mm.3D_glcm_DifferenceEntropy | log |  | 0.06 | 1.00 |
| log.sigma.2.5.mm.3D_glcm_JointEnergy | log |  | 0.05 | 1.00 |
| log.sigma.2.5.mm.3D_glrlm_ShortRunHighGrayLevelEmphasis | log |  | 0.07 | 1.00 |
| log.sigma.3.5.mm.3D_glrlm_RunLengthNonUniformity | log |  | 0.06 | 0.99 |
| log.sigma.3.5.mm.3D_glszm_LowGrayLevelZoneEmphasis | log |  | 0.06 | 1.00 |
| log.sigma.4.5.mm.3D_glszm_SizeZoneNonUniformityNormalized | log |  | 0.08 | 0.98 |
| log.sigma.4.5.mm.3D_glszm_ZoneVariance | log |  | 0.07 | 0.99 |

glcm, gray level co-occurrence matrix; gldm, Gray Level Dependence Matrix; ngtdm, neighbouring gray tone difference matrix; glrlm, gray level run length matrix; glszm, gray level size zone matrix; Imc1, Informational measure of correlation 1; Idn, Inverse difference normalized.
